# Toddler foods and milks don’t stack up against regular foods and milks

**DOI:** 10.1186/s12937-022-00765-1

**Published:** 2022-02-25

**Authors:** Jennifer McCann, Kelsey Beckford, Holly Beswick, Melanie Chisholm, Julie Woods

**Affiliations:** 1 Institute for Physical Activity and Nutrition (IPAN), School of Exercise and Nutrition Sciences, Deakin University, Geelong, Australia; 2 Deakin University, Geelong, Australia; 3 VACCHO, Collingwood, Australia; 4 Victorian Department of Health, Melbourne, Australia; 5 Institute for Physical Activity and Nutrition (IPAN), School of Exercise and Nutrition Sciences, Geelong, Australia

**Keywords:** Toddler, Public health, Nutrition, Cost

## Abstract

**Aim:**

To compare the cost and nutritional profiles of toddler-specific foods and milks to ‘regular’ foods and milks.

**Methods:**

Cross-sectional audit of non-toddler specific (‘regular’) foods and milks and secondary analysis of existing audit data of toddler specific (12-36 months) foods and milks in Australia.

**Main findings:**

The cost of all toddler-specific foods and milks was higher than the regular non-toddler foods. Foods varied in nutritional content, but toddler foods were mostly of poorer nutritional profile than regular foods. Fresh milk cost, on average, $0.22 less per 100 mL than toddler milk. Toddler milks had higher mean sugar and carbohydrate levels and lower mean protein, fat, saturated fat, sodium and calcium levels per 100 mL, when compared to fresh full fat cow’s milk.

**Conclusions:**

Toddler specific foods and milks cost more and do not represent value for money or good nutrition for young children.

## Background

Late infancy and early toddlerhood are key times for young children to be exposed to a variety of food textures, flavours and tastes [1–3]. Children are pre-disposed to have a preference for sweet and salty tastes, reject sour and bitter flavours, and reject new foods [4, 5]. Young children learn to eat what is provided through flavour and texture familiarisation [4], and if unhealthy eating habits are formed in the early years, they are likely to take these into adulthood and be at greater risk of diet related chronic diseases [6, 7].

A major shift in the global food system has occurred with ultra-processed foods (UPF) becoming the main source of energy in both adults and children [8–13], in a typical western diet. This is a key driver of the obesity epidemic worldwide [14]. Global research indicates that toddlers are becoming regular consumers of commercially available foods and milks [15, 16]. An Australian study found that discretionary foods contributed just over 10% of total energy intake in toddlers [15], and packaged infant and toddler snack foods made up 9% of this total. Toddler milk consumption is also on the rise, with Australian research demonstrating close to 40% of toddlers are consuming toddler milks [16], and this is predicted to continue to rise [9, 17]. In addition, research has shown that the largely unregulated retail market for toddler foods and milks is increasing at unprecedented rates around the globe [9, 18].

Despite Australian research demonstrating that a healthy diet (low in UPF and drinks) costs less than an unhealthy diet (high in UPF and drinks) [19], convenience [20], on-pack marketing [21–23] traditional marketing such as television, radio and print [24, 25], as well as social media [26] are strongly influencing consumers to choose unhealthy diet patterns (typically high in UPF and drinks) [27]. As family lives become busier, consumer demand for convenience is strong, despite the cost [20, 28, 29]. Planning a healthy diet takes time [20] which is in short supply for many families, and they may compromise health for convenience to fit busy lifestyles [28, 30]. Food cost is an important determinant of food intake and of food security, with those in lower socioeconomic positions more likely to experience food insecurity [31] and have dietary patterns that are generally lower in the basic healthy foods such as fresh fruit and veg and higher in discretionary, UP foods [32]. Dietary intake studies from the US have reported that up to 8% of total dietary intake for toddlers in low-income households comes from added sugars, compared to 2% in higher income households, and the excess sugar, sodium, saturated fat in toddlers’ diets is mostly from commercial foods and drinks [33].

As many toddler specific foods and milks are promoted on pack as being healthy, better than homemade, and even necessary, and being mindful of nutrition equity issues with food costs, an analysis was conducted to compare the cost and nutritional profiles of toddler specific foods and milks to non-toddler foods and milks.

## Methods

Using the online shopping sites of Coles and Woolworths (representing approximately 70% of the market share of all grocery sales in Australia) [34], a search was conducted in September 2020 to collect data on the cost and nutrition of ‘regular’ foods and milks (full fat milks, cereal/fruit bars, dried and fresh fruit, raw ingredient balls, rice crackers and yoghurts, across a range of cost values representing the least expensive, regular price brands and premium brands). These were compared to all category specific, equivalent toddler foods and milks (marketed towards children aged 12-36 months) identified and included in a 2019 Australian audit [35]. Prices of the toddler foods and milks were checked in 2020 and updated if required if they differed from the 2019 audit results. Data pertaining to the country of origin, unit price, unit size (g), suggested serving size and number of serves per pack were collected from the product images and descriptions, and price per serve was calculated. Nutrient content per serve was determined using the nutrition information panel. Nutrients assessed were energy (kJ), protein (g), fat (g), saturated fat (g), carbohydrates (g), total sugars (g), sodium (mg) (and calcium (mg)) for milk, yoghurts, and toddler milks and yoghurts only. Mean cost and nutrient values were calculated for each product category including within categories where regular and premium type products were identified, and subsequent comparisons were then made between the ‘regular’ foods and milks and the toddler specific foods and milks. Regular full fat milks (*n* = 2) were compared with regular toddler milks (*n* = 8), as well as with premium toddler milks (*n* = 12). Premium full fat milks (includes branded (non-home brand) and A2 milks, *n* = 9) were compared with premium toddler milks (*n* = 12), and premium and organic full fat milks (*n* = 13) were compared to premium, A2 and organic toddler milks (*n* = 18).

## Results

There were 88 toddler-specific foods and 26 toddler milks included for comparison to 49 ‘regular’ foods and 15 milks. The majority of toddler snacks (64%) and milks (82%) were manufactured in Australia and New Zealand, with 28% of snacks and 15% of milks imported from Europe and small percentages from Asia and the USA.

Overall, all fresh ‘regular’ milk varieties cost less than their toddler milk counterparts per 100 mL (Tables 1 and 2). Serve sizes for all ‘regular’ foods were larger than serve sizes for toddler-specific snack foods, thus the analysis for comparisons were conducted using per 100 g/mL for consistency. Whilst serve sizes for toddler milks were lower than for ‘regular’ milks, when based on an average toddler milk serve size of 200 mL, there is a cost difference of 44 cents per serve. If one (200 mL) serving of toddler milk was consumed every day for 1 month instead of consuming one serve of full fat cow’s milk, the cost difference would be on average $13 over 1 month and close to $160 a year. This is based on the combined mean price of all toddler milks, with premium milks costing more, and regular toddler milks (non-premium) costing less, with a range of $8-$17/month, equating to $100-$208/year. Furthermore, the fresh milks had higher mean protein, fat, saturated fat, and sodium levels and lower mean carbohydrate and total sugar levels per 100 mL, when compared to toddler milks. Some toddler milks had 8 g more sugar per 200 mL serve than fresh full fat milks, equating to 60 teaspoons per month, or close to 3 kg of sugar per year if one serve was consumed daily when compared to fresh full milk. The mean calcium levels varied across toddler milks, but the mean calcium level of all toddler milks was lower than the mean levels in all fresh full fat milks. The premium stage 4 and premium organic toddler milks had significantly higher mean calcium levels per 100 mL than any other toddler milks or fresh full fat milks.

**Table 1:** Mean (± SD) cost and nutritional data for fresh full fat milk and toddler milks

Product category	***n***=	Price per 100 mL	Energy per 100 mL (kj)	Protein per 100 mL (g)	Total fat per 100 mL (g)	Sat fat per 100 mL (g)	Carbs per 100 mL (g)	Total sugar per 100 mL (g)	Sodium per 100 mL (mg)	Calcium per 100 mL (mg)
**Full fat fresh Milks**										
**Regular fresh full fat milk (Home brand)**	**2**	**$0.13 (0)**	**281 (16)**	**3.5 (0)**	**3.5 (0.1)**	**2.2 (0.1)**	**5.5 (1.1)**	**5.5 (1.1)**	**36 (1.4)**	**114 (9.9)**
Branded full fat milk	7	$0.19 (0.04)	278 (17)	3.3 (0.1)	3.8 (0.5)	2.4 (0.3)	4.8 (0.2)	4.8 (0.2)	44.9 (5.4)	119.2 (5.4)
A2 full fat milk	2	$0.25 (0.03)	267 (11)	3.4 (0.1)	3.5 (0.1)	2.4 (0.07)	4.7 (0.4)	4.6 (0.2)	38.5 (7.8)	113.5 (6.4)
**Premium fresh full fat milk**^**a**^	**9**	**$0.20 (0.04)**	**276 (16)**	**3.3 (0.1)**	**3.7 (0.4)**	**2.4 (0.3)**	**4.8 (0.2)**	**4.8 (0.2)**	**43.4 (6.1)**	**117.8 (5.8)**
Organic full fat milk	4	$0.28 (0.06)	288 (20)	3.5 (0.2)	3.9 (0.3)	2.5 (0.3)	4.7 (0.1)	4.6 (0.3)	49.5 (8.6)	123 (4.6)
**Premium**^**a**^ **+ Organic fresh full fat milk**	**13**	**$0.22 (0.06)**	**280 (17)**	**3.4 (0.2)**	**3.8 (0.4)**	**2.4 (0.3)**	**4.8 (0.2)**	**4.7 (0.2)**	**45.3 (7.3)**	**119.5 (5.8)**
**All fresh milks**	**15**	**$0.21 (0.06)**	**280 (17)**	**3.4 (0.2)**	**3.7 (0.4)**	**2.4 (0.3)**	**4.9 (0.4)**	**4.8 (0.5)**	**44.1 (7.5)**	**118.7 (6.3)**
**Toddler milks**										
Regular stage 3 (12mo+)	6	$0.32 (0.12)	240 (84)	1.93 (0.5)	2.59 (0.3)	0.96 (0.4)	9 (0.8)	7.01 (1.4)	22.57 (4.2)	103.94 (7.7)
Regular stage 4 (24mo+)	2	$0.28 (0)	283 (8)	1.8 (0.6)	2.47 (0.5)	1.03 (0.7)	9.07 (0.9)	7.32 (1.6)	22.5 (3.5)	105 (7.1)
**All regular**	**8**	**$0.31 (0.1)**	**251 (74)**	**1.9 (0.5)**	**2.56 (0.3)**	**0.98 (0.4)**	**9.01 (0.8)**	**7.09 (1.4)**	**22.55 (3.8)**	**104.21 (7)**
Premium stage 3 (12mo+)	10	$0.51 (0.44)	286 (15)	2.3 (0.6)	2.74 (0.4)	1.15 (0.4)	8.41 (1)	6.81 (1.6)	27.25 (6.3)	106.45 (15.2)
Premium stage 4 (24mo+)	2	$0.32 (0.04)	273 (28)	1.8 (1)	2.1 (0.1)	0.8 (0.4)	16.1 (9.8)	9.4 (0.9)	37.5 (14.9)	173 (103.2)
**All premium non-A2/Organic**	**12**	**$0.48 (0.41)**	**284 (16)**	**2.21 (0.6)**	**2.63 (0.4)**	**1.08 (0.4)**	**9.69 (4.3)**	**7.24 (1.8)**	**28.96 (8.3)**	**117.54 (42.8)**
Premium A2	3	$0.47 (0.07)	287 (5)	1.97 (0.6)	2.97 (0.1)	0.83 (0.2)	8.35 (0.4)	7.68 (1)	27.33 (5.5)	92.67 (10.6)
Premium Organic	3	$0.54 (0.11)	292 (21)	2.7 (0.9)	3.2 (0.8)	1.43 (0.8)	7.57 (1.9)	6.77 (1.7)	32.5 (9)	124.67 (29.7)
**All toddler formulas**	**26**	**$0.43 (0.3)**	**275 (44)**	**2.14 (0.6)**	**2.71 (0.5)**	**1.06 (0.5)**	**9.08 (3)**	**7.19 (1.5)**	**27.21 (7.4)**	**111.39 (31.6)**

^a^Premium fresh milk included branded (non-home brand) and A2 varieties

**Table 2:** Difference in cost and nutrition of toddler foods and milks to ‘regular’ foods and milks

	Price per 100 mL/g	Energy per 100 ml/g (kJ)	Protein per 100 mL/g (g)	Total fat per 100 mL/g (g)	Sat Fat per 100 mL/g (g)	Carbohydrate per 100 mL/g (g)	Total Sugar per 100 mL/g (g)	Sodium per 100 mL/g (mg)	Calcium per 100 mL/g (mg)
**Regular fresh full fat milk vs regular toddler milk**	$0.14^b^	28.01	1.46	1.13	1.39	4.05^b^	2.12^b^	20.34	13.67
**Regular fresh full fat milk vs premium toddler milk**	$0.31^b^	5.21^b^	1.14	1.06	1.28	4.72^b^	2.27^b^	13.93	0.33
**Premium fresh full fat milk vs premium toddler milk**	$0.29^b^	5.67^b^	1.10	1.13	1.33	4.87^b^	2.42^b^	15.90	1.62
**Premium & organic fresh full fat milk vs premium & organic toddler milk**	$0.08	6.36^b^	1.13	0.96	1.32	4.35^b^	2.52^b^	16.03	4.91
**All milk vs all formula**	$0.22^b^	4.55	1.26	1.0	1.33	4.22^b^	2.37^b^	16.86	7.32
**Cereal/fruit bars vs Toddler cereal/fruit bars**	$2.21^b^	113.93	0.08^b^	2.23	2.24	2.14	2.98	74.59^b^	
**Dried fruit vs Dried fruit snacks**	$9.94^b^	132.97^b^	0.12^b^	0.45	0.09	7.22^b^	0.72^b^	48.09	
**Fresh fruit vs Fruit based snacks**^**a**^	$11.38^b^	799.76^b^	3.20^b^	2.69^b^	0.59^b^	38.66^b^	31.46^b^	34.93^b^	
**Organic/Raw balls/bites vs Raw/organic snacks**	$0.69^b^	49.50	4.79	0.59^b^	6.53^b^	2.60^b^	4.85^b^	10.55	
**Rice crackers vs Rice based snacks**	$8.32^b^	63.85^b^	0.86	3.23^b^	1.19^b^	3.59	3.24^b^	211.34	
**Yoghurt vs Toddler yoghurts**	$1.31^b^	62.09	0.75	0.68^b^	0.42^b^	4.41	4.89	11.58	27.73^b^

^a^Fruit based snacks included only snacks which were primarily fruit, such as purees, fruit chews and fruit buttons

^b^Toddler product greater than ‘regular’ product, Premium fresh milk included branded (non-home brand) and A2 varieties

When comparing the toddler-specific snacks and yoghurts (labelled for within the age range of 12-36 months) to similar ‘regular’ products on the market, fruit-based snacks had a higher mean total sugar compared to regular fresh fruit per 100 g (40 g and 9.2 g respectively) and the fresh fruit had lower mean values for all nutrients on the nutrition information panel when compared to the toddler-specific fruit sweetened snacks per 100 g. ‘Regular’ rice-based snacks had lower mean total and saturated fat and total sugar per 100 g, compared to the toddler-specific varieties, in addition to higher mean sodium per 100 g. Toddler-specific yoghurts had lower mean protein, carbohydrate, total sugar, sodium, and calcium per 100 g than ‘regular’ yoghurts. Mean sodium levels per 100 g were nearly three-times lower in toddler rice-based snacks and close to six-times lower in dried fruit when compared to ‘regular’ products, although there is a high degree of variability in sodium levels in all toddler and regular foods, suggesting that consumer choice would be important in purchasing the lowest sodium items (Tables 2 and 3).

**Table 3:** Mean (SD) cost and nutritional data for ‘regular’ foods and toddler food categories

Product category	***n***=	Price/100 g	Energy/ 100 g (kJ)	Protein/ 100 g (g)	Total fat/ 100 g (g)	Saturated fat/ 100 g (g)	Carbohydrate/ 100 g (g)	Total sugar/ 100 g (g)	Sodium/ 100 g (mg)	Calcium/ 100 g (mg)
Cereal/fruit bars	8	$1.43 (0.76)	1628 (146)	5.8 (2.2)	10.3 (6.7)	5.2 (4.9)	64.2 (9.8)	31 (16.4)	52.1 (55.5)	
Dried fruit	7	$1.37 (0.7)	1281 (100)	2.3 (0.9)	0.9 (1)	0.2 (0.2)	68.1 (5.8)	61.7 (6.6)	58. (53.8)	
Fresh fruit	10	$1.08 (1.43)	214 (81)	0.7 (0.4)	0.1 (0.1)	0 (0)	10 (4.5)	9.2 (3.25)	0.9 (1.1)	
Organic/Raw balls/bites	5	$4.86 (0.81)	1750 (131)	11.1 (10)	16.1 (4.3)	4.7 (4)	53.3 (14.7)	36.5 (15.6)	79 (123.9)	
Rice crackers	9	$1.71 (1.54)	1761 (106)	7.5 (0.9)	7.2 (5.6)	3.1 (4.4)	79.2 (6.9)	7.8 (11.9)	324.5 (145.6)	
Yoghurt	10	$0.7 (0.44)	400 (46)	4.8 (0.5)	2.6 (1.1)	1.7 (0.8)	12.7 (1.6)	11.4 (1.6)	57.2 (7.9)	196.7 (54)
Cereal/fruit bars	14	$3.64 (1.87)	1514 (115)	5.9 (0.9)	8.1 (2.1)	2.9 (1.4)	62 (9.8)	34 (5.9)	126.7 (99.1)	
Dried fruit	5	$11.31 (8.26)	1414 (111)	2.4 (0.9)	0.5 (0.1)	0.1 (0)	75.3 (4.2)	62.4 (10.8)	10.2 (5.5)	
Snack - fruit sweetened	11	$12.46 (10.53)	1013 (635)	3.9 (5.2)	2.8 (5.1)	0.6 (0.9)	48.7 (30.6)	40.6 (27.1)	35.8 (66)	
Organic/Raw balls/bites	12	$5.54 (1.21)	1701 (231)	6.4 (2.7)	16.7 (9.8)	11.2 (8.4)	55.9 (8.7)	41.3 (8.2)	68.5 (92.1)	
Rice crackers	16	$10.03 (4.58)	1825 (190)	6.7 (1)	10.5 (8.5)	4.3 (5.6)	75.6 (11)	11 (10.5)	113.2 (123.2)	
Yoghurt	26	$2.01 (1.14)	338 (34)	4 (0.4)	3.3 (1)	2.2 (0.7)	8.3 (1.6)	6.5 (1.7)	45.6 (8.5)	169 (14)

The average price of 1 kg of fresh fruit (average cost of banana, apple, pear, strawberries, raspberries, orange and watermelon at the time of analysis in September 2020) was $10.80 whereas the average price per kg of toddler fruit-based snacks was $124.60 (around 12 times more expensive). Similarly, the average price of 1 kg of dried fruit was $13.70 and the average price of 1 kg of toddler dried fruit was $113.10 (around 10 times more expensive). Yoghurt marketed specifically for toddlers was approximately $1.31 more expensive per 100 g than regular yoghurt, equating to around $40 per month, or $470 per year if one 100 g serving was consumed daily.

## Discussion

This study has demonstrated that toddler specific foods and milks cost more and do not represent value for money or good nutrition. There is limited research on consumption of toddler specific packaged foods and toddler milks in Australia and globally. The evidence that does exist includes two Australian studies on children aged 12-24 months [36, 37]. These studies reported that consumption of packaged foods is common with an average of 60% of toddlers consuming commercial sweet foods. The studies also reported 48% of toddlers consumed yoghurt (plain, flavoured and custard), 20% consumed dried fruit, 23% consumed sugar and sugary products and 35% consumed discretionary cereal products daily (based on 24 h recall and food-frequency questionnaires). Additional Australian research reported around 36% of toddlers consume toddler milk daily [16], and Australian national health survey data has also shown that close to 40% of toddlers aged 2-3 years consume yoghurt daily and close to 50% consume confectionery and cereal/nut/fruit/seed bars daily [38].

Putting the above consumption patterns into context with our results, it can be deduced that if a toddler were to consume the toddler-specific products daily, the cost would be much higher than if the ‘regular’ version of the product was purchased. This equates to a larger outlay of money (e.g. $40 extra per month for one 100 g toddler-specific yoghurt daily, and $13 more per month for one 200 mL serve of toddler milk), and with the cost of living increasing and wage growth stagnating [31], this impacts the household budget, and may become a social equity issue.

The same calculations can be made for all the toddler-specific products, and all lead to the same conclusion: toddler-specific packaged foods cost significantly more than ‘regular’ foods. Looking at the nutrition provided from the toddler-specific foods, these vary greatly across the product categories. As an example, toddler-specific rice crackers had on average per 100 g, lower mean protein, carbohydrate and sodium than the ‘regular’ rice crackers, but they also contained on average per 100 g, higher mean total fat, saturated fat, total sugar and energy. So, while a toddler rice cracker may seem like it is healthier as it is lower in sodium, it is a trade-off, as it is higher in sugar. Low sodium ‘regular’ rice crackers (which are classified as core in the Australian Dietary Guidelines) are present in small numbers in the Australian retail market, and they do have lower levels of sodium and sugar per serve and per 100 g than even a toddler-specific rice cracker, and may be the most suitable option for a rice-based snack for all ages, but would still not be recommended if they are UP.

Following on from this, if the example of toddler milk is examined in more detail, if a toddler milk was consumed daily, the toddler milks will ultimately provide more micronutrients than ‘regular’ milk due to fortification, but are an UPF. However, if the NIP nutrients mandated to be present are focused on, toddler milks are actually higher in carbohydrate, total sugar and energy per 100 mL than ‘regular’ milks, and the higher levels of these nutrients in the toddler milk are not required or recommended for healthy toddlers, as stated by the World Health Organization [39, 40] and the National Health and Medical Research Council [41].

A strength of this study is that the products included represent a relatively complete audit of all toddler-specific products on the market at the time and ‘regular’ foods were matched as closely as possible and are from the two leading supermarket outlets in Australia. A limitation of this study is that toddler-specific food consumption data is scarce however, we have included the most relevant data where possible. This indicates that consumption is considerable and that therefore there is a likely impact to food budgets if these products are chosen over regular foods. We are also not suggesting that the regular food alternatives are healthier choices than the toddler specific foods – indeed many are ULP and discretionary themselves. However, the point remains that a specific market has been created for toddler foods that demand a higher price premium.

## Conclusion

To ensure that young children have a healthy start to life and become familiar with healthy foods (non UPF and drinks) from a young age, the message here is to buy regular family foods for better nutrition and cost savings, and avoid relying on toddler-specific foods and milks. Toddler milks and toddler-specific packaged foods are expensive distractions from what should actually be a simple, healthy diet. To support this, a strong regulatory approach to the marketing and nutrient content of toddler-specific food and milks is needed.

## Data Availability

The datasets used and/or analysed during the current study are available from the corresponding author on reasonable request.

## Abbreviations

UPF: Ultra-processed foods
UP: Ultra-processed
